# Lipid damage is the best marker of oxidative injury during the cardiac remodeling process induced by tobacco smoke

**DOI:** 10.1186/s40360-018-0268-4

**Published:** 2018-11-16

**Authors:** Maria Angélica Martins Lourenço, Mariana Gobbo Braz, Aline Garcia Aun, Bruna Letícia Buzati Pereira, Fábio Henrique Fernandes, Elisa Moya Kazmarek, Tatiana Fernanda Bachiega, Silmeia Garcia Zanati, Paula Schmidt Azevedo, Bertha Furlan Polegato, Ana Angélica Henrique Fernandes, Sergio Alberto Rupp de Paiva, Leonardo Antonio Mamede Zornoff, Marcos Ferreira Minicucci

**Affiliations:** 1 Internal Medicine Department, Botucatu Medical School, UNESP - São Paulo State University, Botucatu, Brazil; 2 Department of Anesthesiology, Botucatu Medical School, UNESP - São Paulo State University, Botucatu, Brazil; 3 Department of Genetics, Institute of Biological Sciences, UNESP - São Paulo State University, Botucatu, Brazil; 4 Chemistry and Biochemistry Department, Institute of Biological Sciences, Botucatu Medical School, UNESP - São Paulo State University, Botucatu, Brazil; 5 Departamento de Clínica Médica, Faculdade de Medicina de Botucatu, Rubião Júnior s/n, Botucatu, SP CEP: 18618-000 Brazil

**Keywords:** Comet assay, DNA damage, Protein carbonyl groups, Cigarette smoke

## Abstract

**Background:**

Oxidative stress is one potential mechanism that explain the direct effects of smoking on cardiac remodeling process. However, no study has compared different myocardial products of macromolecule oxidation after tobacco smoke exposure. Thus, the aim of this study was to investigate the lipid hydroperoxide (LH) levels, protein carbonyl concentrations and DNA damage in cardiac tissue of rats exposed to tobacco smoke.

**Methods:**

Male Wistar rats were divided into two groups: group C (control, *n* = 14) composed of animals not exposed to cigarette smoke; group ETS (exposed to tobacco smoke, n = 14) composed by animals exposed to cigarette smoke. The animals were exposed to 2 month of ETS and morphological, biochemical and functional analyses were performed.

**Results:**

Cardiac cotinine levels were elevated in the ETS group. In addition, the myocyte cross-sectional area was higher in the ETS group. (C = 266.6 ± 23.2 μm^2^ and ETS = 347.5 ± 15.1 μm^2^, *p* <  0.001). Cardiac LH was higher in the ETS group than in group C (C = 196.4 ± 51.5 nmol/g and ETS = 331.9 ± 52.9 nmol/g, p <  0.001). However, there were no between-group differences in cardiac protein carbonyl concentration or DNA damage.

**Conclusions:**

Therefore, our results suggest that, in this model, lipid damage is a good marker of oxidative damage during the cardiac remodeling process induced by 2 months of exposure to tobacco smoke.

## Background

Tobacco smoke is one of the major risk factors and is the leading cause of preventable death for cardiovascular disease [1]. In addition to the well-known effects of cigarette smoke on vascular systems, the direct effects of smoking on cardiac remodeling have been studied [2–7]. Using an animal model, other have found that exposure to tobacco smoke (ETS) induces enlargement of the ventricular chambers and is associated with myocardial hypertrophy, as well as cardiac dysfunction [2–9]. Potential mechanisms for these alterations include hemodynamic and neurohormonal changes, mitogen-activated protein kinase activation, cardiac lipotoxicity and oxidative stress [5–7, 10–13].

Oxidative stress occurs when there is an imbalance between reactive oxygen species (ROS) production and antioxidant systems. Tobacco smoke has more than 4000 chemical compounds and 10^15^ to 10^17^ free radicals that cause tissue inflammation and ROS production [14]. However, the importance of ROS is not restricted to oxidative damage. In nanomolar concentrations, ROS play an important role in signal transduction pathways and in the modulation of transcription factors [15, 16]. There are different sources of ROS, including mitochondrial electron transport, the cytochrome P450 system and the nicotinamide adenine dinucleotide phosphate (NADPH) oxidase system [15, 16]. We have previously shown that exposure of rats to tobacco smoke for two months increases myocardial NADPH activity, decreased the levels of antioxidant enzymes and increased cardiac lipid hydroperoxide levels [5].

Oxidative stress can be measured by different products derived by damage to proteins, lipids and DNA. Of note, clinical and experimental studies showed different results regarding biomarkers of oxidative damage. 8-hydroxyguanine, a biomarker of oxidative DNA damage was increased in human leukocytes, urine and lung tissue of smokers compared to nonsmokers [17–19]. However, other clinical and experimental studies showed no difference in DNA damage, assessed by the comet assay, in peripheral blood of human or rats exposed to tobacco smoke [20–22]. Likewise, plasma protein carbonyl concentration, a marker of protein oxidative damage, was increased in heavy smokers in some but not all studies [19, 23].

The different concentrations of these by-products of macromolecule oxidation could be due to the time of their measurement, tissue specificity or the nature of the ROS [24, 25]. To the best of our knowledge, no study has compared different myocardial products of macromolecule oxidation after tobacco smoke exposure. We decided to study these products following 2 months of ETS, because this time is sufficient for the development of alterations in cardiac morphology and function. Since others have shown that 2 months of ETS exposure is associated with change in ventricular function and structure, the aim of the study was to determine the cardiac levels of lipid hydroperoxide, protein carbonyl concentration and DNA damage in rats exposed to tobacco smoke.

## Methods

All experiments and procedures were performed in accordance with the National Institute of Health Guide for the Care and Use of Laboratory Animals and were approved by the Animal Ethics Committee of Botucatu Medical School (1116–2015).

Male Wistar rats weighing 200 g to 250 g were purchased from the Laboratory Animal Center of Botucatu Medical School. The rats were divided into 2 groups: group C (control, *n* = 14) composed by animals not exposed to cigarette smoke; group ETS (exposed to tobacco smoke, n = 14) composed by animals exposed to cigarette smoke. Food and water were supplied ad libitum. The animals were observed during 2 months, during which morphological, biochemical and functional analyses were performed. The rats were sacrificed with thiopental (80 mg/kg, IP), after which blood samples and heart were collected.

### Exposure to tobacco smoke

The ETS rats were exposed to cigarette smoke in a chamber (dimensions 95x80x65 cm) connected to a smoking device constructed based on a model published by Wang et al. and adapted by Paiva et al. [8, 26]. During the first week, the number of cigarettes was gradually increased from 5 to 10 cigarettes delivered in a 30 min period, administered twice each afternoon. Subsequently, 10 cigarettes were used in each smoking trial, repeated four times/day, twice in the morning and twice in the afternoon. The cigarette composition per unit is: 0.8 mg of nicotine; 10 mg of tar; and 8 mg of carbon monoxide. In previous studies, we analyzed carboxyhemoglobin level in smoking rats. Given the corresponding carboxyhemoglobin levels, this protocol is similar to 3–4 pack/day in a human. On the other hand, this protocol did not result in hypoxia [2].

### Isolated heart study: langendorff preparation

After 2 months of exposure, six animals from each group were anesthetized with thiopental (80 mg/kg, IP) and received unfractionated heparin (2000 I.U., IP). After sternotomy, the rats were artificially ventilated, and the ascending aorta was dissected and cannulated. Retrograde perfusion was initiated with a modified Krebs-Henseleit solution, constantly gassed with a mixture of 95% O_2_ and 5% CO_2_, and a perfusion pressure of 75 mmHg [27]. We performed the aorta cannulation with the heart in situ. With this procedure, we reduce the ischemic time. Only after aorta cannulation, and retrograde perfusion initiation we removed the heart. The heart was removed and transferred to an isolated heart perfusion apparatus (size 3, type 830, Hugo Sachs Elektronik - March-Hugstetten, Germany). A balloon was inserted in the left ventricular cavity, and the volume inside the balloon was modified to obtain a diastolic pressure of zero at 25 mmHg. We registered the diastolic and systolic pressures, the maximum left ventricular pressure decrease rate (-dP/dt) and the maximum left ventricular pressure development rate (+dP/dt). Systolic function was evaluated by +dP/dt, and diastolic function by -dP/dt. Developed pressure was also obtained.

The hearts that were submitted to the isolated heart study were not utilized for any other analysis as retrograde perfusion can interfere with subsequent biochemical analysis.

### Morphometric analysis

The right and left ventricles (including the interventricular septum) were dissected and separated. Transverse sections of the LV were fixed in 10% buffered formalin and embedded in paraffin. Five-micron-thick sections were stained with hematoxylin and eosin (HE). The myocyte cross-sectional area was determined for a minimum of 50 myocytes per HE-stained cross section. The measurements were obtained from digitized images (× 400 magnification) collected using a video camera attached to a Leica microscope and computerized image analysis software (Image-Pro Plus 3.0, Media Cybernetics; Silver Spring, MD). The cells selected for analysis were transversely cut with the nucleus clearly identified in the center of the myocyte [5].

### Cardiac lipid hydroperoxide and protein carbonyl concentrations

Two hundred-milligram samples of the left ventricle were homogenized in 5 mL 0.1 M cold sodium phosphate buffer, pH 7.4, containing 1 mM ethylenediaminetetraacetic acid (EDTA). Lipid hydroperoxide (LH) was measured through the hydroperoxide-mediated oxidation of Fe^2+^ as previously published [28–30]. The spectrophotometric determinations were performed in a Pharmacia Biotech spectrophotometer with a temperature-controlled cuvette chamber (U*V*/*v*isible Ultrospec 5000 with Swift II Applications software connected to computer system control, 974,213, Cambridge, England, UK). All reagents were from Sigma (St. Louis, Missouri, USA) [28–30].

Protein carbonyl was analyzed through reaction with dinitrophenylhydrazine and formation of a Schiff base, according to the method described by Reznick and Packer [31]. The level of protein carbonyl was quantified spectrophotometrically at 360 nm using an extinction coefficient of 22,000 M^-1.^cm^− 1^.

### DNA damage evaluation

The Comet assay detects DNA damage at the individual cell level. Therefore, cardiomyocytes from LV fragments were isolated as described by Pool-Zobel et al. [32]. Suspensions of 2–6 × 10^6^ cells were obtained per fragment. The cell viability was evaluated by the FDA/EtBr assay according to Strauss [33]. Briefly, a fresh staining solution was prepared containing 30 μL FDA in acetone (5 mg/mL), 200 μL EtBr in DPBS (200 μg/mL), and 4.8 mL DPBS. Then, 25 μL from staining solution was mixed with 25 μL of cardiomyocyte suspension, spread onto a slide, and covered with a coverslip. Green-fluorescent color indicated viable cardiomyocyte, whereas red-stained nuclei indicated dead cardiomyocytes. At least 200 cells were counted per sample.

The alkaline Comet assay measures single and double strand breaks, labile sites (SBs) and apurinic/apyrimidinic (AP) sites. DNA damage, including SBs, AP sites, oxidased pyrimidines (endonuclease III-sensitive sites), and altered purines [sites sensitive to formamidopyrimidine glycosylase (FPG)], was detected by the alkaline comet assay modified with the lesion-specific enzymes, FPG and endo III [34]. All procedures were conducted in the dark to minimize spurious sources of DNA damage. Briefly, 15 μL of the cardiomyocyte suspensions were embedded into 0.5% low-melting point agarose (Sigma) and spread on agarose precoated microscope slides. Six slides were prepared for each sample. Slides were immersed overnight in freshly prepared cold lysing solution (2.5 M NaCl, 100 mM EDTA, 10 mM Tris, 1% sodium salt N-Lauryl sarcosine, pH 10.0, with 1% Triton X-100 and 10% DMSO added fresh) at 4 °C. After lysing, the slides were washed three times in enzyme buffer and then two slides for each treatment were incubated at 37 °C for 45 min with 100 μL of endo III (1:1000) or 100 μL of FPG (1:1000) or with enzyme buffer only. Endo III recognizes oxidized pyrimidines, while FPG identifies oxidized purines, especially 8-oxo-guanine. Enzyme buffer by itself was used to identify SBs. Subsequently, the cells were exposed to alkali buffer (1 mM EDTA and 300 mM NaOH, pH ≅ 13.4), at 4 °C, for 40 min to allow DNA unwinding and expression of alkali-labile sites. Electrophoresis was conducted in the same solution at 4 °C, for 30 min, at 25 V (1 v/cm) and 300 mA. After electrophoresis, the slides were neutralized (0.4 M Tris, pH 7.5), stained with 40 μL EtBr (20 μg/mL), and analyzed by fluorescence microscopy at 400× magnification using an image analysis system (Comet Assay II–Perceptive Instruments, Suffolk, UK). For each rat, 300 cells, i.e.*,* 100 randomly selected cells (50 from each of two replicate slides) from each treatment (FPG, Endo III or enzyme buffer only), were evaluated 100 randomly selected cells (50 from each of two replicate slides) for each treatment (FPG, Endo III or enzyme buffer only) were evaluated and the mean tail intensity was determined. Tail intensity according to Comet Assay II–Perceptive Instruments is defined as “the sum of all intensity values in the tail region less those which are part of the mirrored head region. The value is also expressed as a percentage of the total Comet or cell intensity” [34].

### Evaluation of serum cotinine

Serum cotinine levels were evaluated by ELISA according to the manufacturer’s instructions (Sigma-Aldrich, St. Louis, MO, USA; product #SE120083). The sensitivity of the ELISA kit was 1 ng/ml.

### Statistical analysis

Comparisons between groups were made by Student’s *t* test for parameters with normal distribution. Otherwise, comparisons between groups were made using the Mann-Whitney U test. Data were expressed as the mean ± SD or medians (including the lower quartile and upper quartile). Data analysis was carried out with SigmaStat for Windows v2.03 (SPSS Inc., Chicago, IL). The significance level was considered 5%.

## Results

Serum cotinine levels, as expected, were elevated in the ETS group [C = 0 (0–0) ng/ml and ETS = 2.52 (1.80–3.10) ng/ml, *p* = 0.008]. This result confirms the efficacy of the procedure for exposing animals to cigarette smoke. Isolated heart study data are shown in Table 1. There were no differences in systolic or diastolic functions between the groups.

**Table 1:** Morphological and Isolated heart study data

	C Group *n* = 8	ETS Group *n* = 8	*P* value
LVW/BW (mg/g)	1.94 ± 0.25	2.07 ± 0.16	0.053
RVW/BW (mg/g)	0.48 (0.43–0.55)	0.54 (0.45–0.96)	0.328
Lung weight/BW	3.97 (3.85–4.07)	5.13 (4.82–5.54)	< 0.001
Liver weight/BW	31.1 ± 3.2	30.1 ± 3.2	0.543
-dP/dt (mmHg)^a^	1896 ± 161	1958 ± 129	0.476
+dP/dt (mmHg)^a^	2521 ± 290	2563 ± 190	0.774
Developed pressure (mmHg)^a^	104.6 ± 6.8	108.3 ± 10.5	0.488

*C* control animals, *ETS* animals exposed to tobacco smoke, *BW* body weight, *LVW* left ventricular weight, *RVW* right ventricular weight, *-dP/dt* the maximum left ventricular pressure decrease rate, *+dP/dt* the maximum left ventricular pressure development rate

Data are expressed as the mean ± SD or medians (including the lower quartile and upper quartile)

^a^sample size *n* = 6 for C group and ETS group

**Fig. 1:**
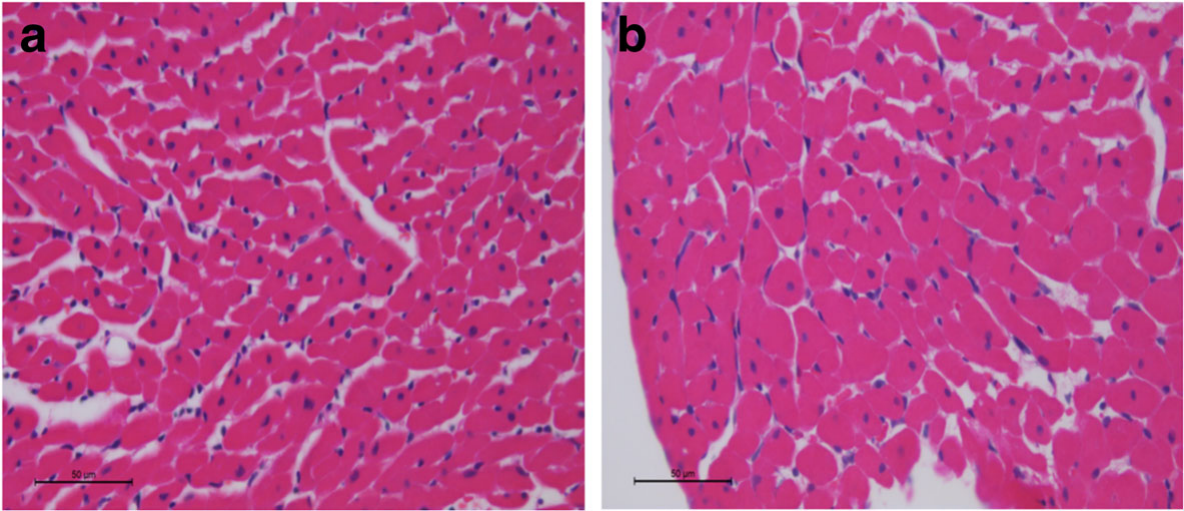
Myocyte cross-sectional area (CSA) **a**. C group. **b** Exposed to Tobacco Smoke group. CSA was higher in ETS group compared to C group

Morphological data are summarized in Table 1. LV and lung weights corrected by body weight (BW) were higher in the ETS group compared with the C group. In addition, the myocyte cross-sectional area (CSA) was higher in the ETS group. (C = 266.6 ± 23.2 μm^2^ and ETS = 347.5 ± 15.1 μm^2^, *p* < 0.001) (Fig. 1).

**Fig. 2:**
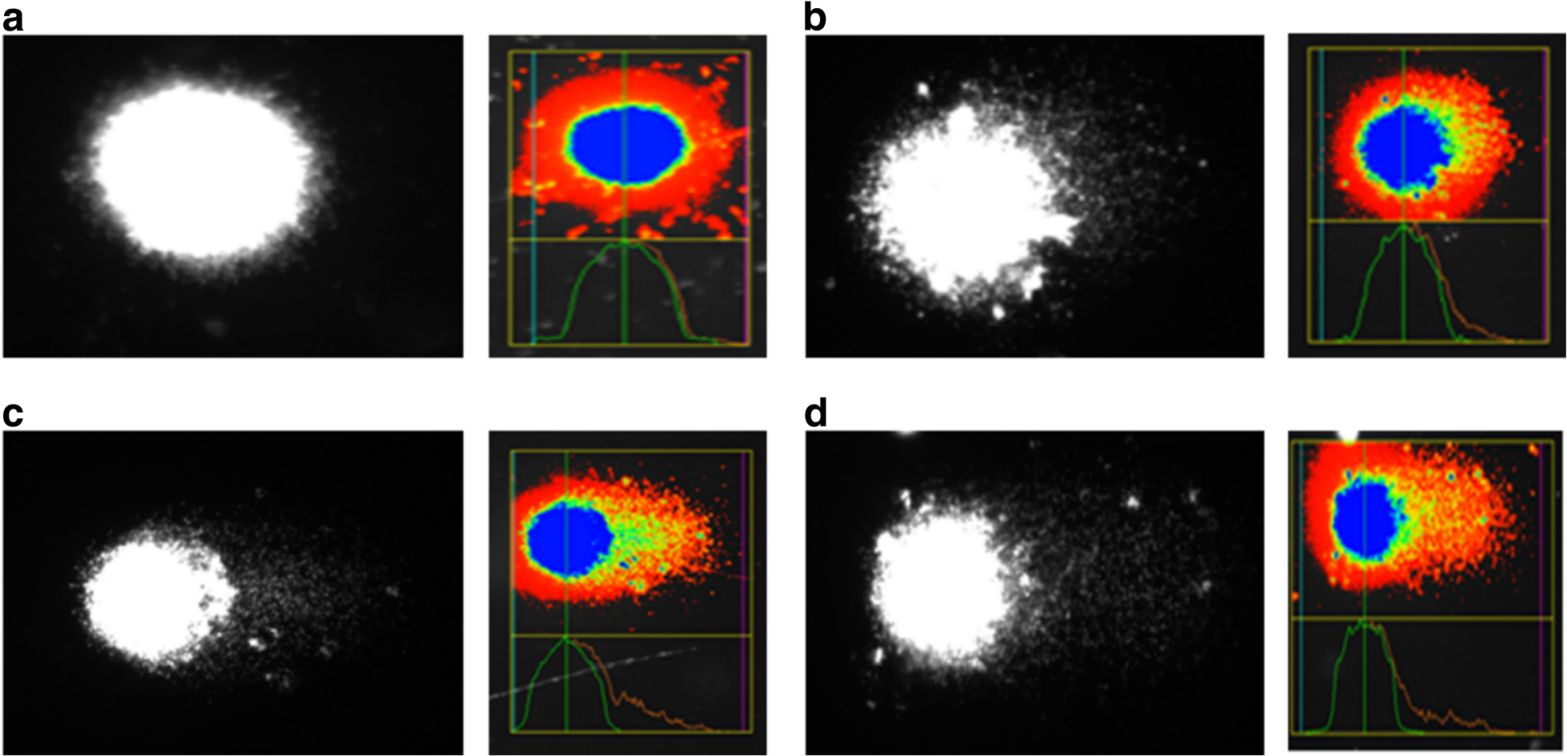
Comet assay. **a** Tail intensity in cardiac tissue from the C group. **b** Tail intensity in cardiac tissue from the ETS group. **c** Tail intensity in blood from the **c** group **d.** Tail intensity in blood from the ETS group. There were no differences between groups in the mean tail intensity in the blood or in the cardiac tissue

The protein carbonyl and LH concentrations are shown in Table 2. The cardiac lipid hydroperoxide was higher in the ETS group compared with group C. However, there was no difference in protein carbonyl concentration between groups. In comet assays, including assays where FPG or endo III were added, there were no differences between groups in the mean tail intensities. (Table 3) (Fig. 2).

**Table 2:** Cardiac determination of protein carbonyl, lipid hydroperoxide and antioxidant enzyme system

	C Group *n* = 8	ETS Group *n* = 8	*P* value
Protein carbonyl (nmol/mg of protein)	2.52 (1.70–3.18)	3.10 (3.07–3.14)	0.310
LH (nmol/g)	196.4 ± 51.5	331.9 ± 52.9	< 0.001

*C* control animals, *ETS* animals exposed to tobacco smoke, *LH* lipid hydroperoxide

Data are expressed as the mean ± SD or medians (including the lower quartile and upper quartile)

**Table 3:** Comet assay

Tail intensity (%)	C Group *n* = 6	ETS Group *n* = 6	P value
Cardiac Tissue			
Buffer	24.0 ± 10.6	18.9 ± 6.4	0.684
FPG	9.4 ± 4.8	10.4 ± 7.1	0.828
Endo III	6.6 (6.2–12.5)	5.4 (4.8–14.2)	0.400
Blood	22.5 ± 3.3	25.9 ± 8.4	0.485

*C* control animals, *ETS* animals exposed to tobacco smoke, *FPG* formamidopyrimidine glycosylase, *Endo III* endonuclease III

Data are expressed as the mean ± SD or medians (including the lower quartile and upper quartile)

## Discussion

The objective of our study was to analyze the different products of oxidative stress derived by smoking-induced damage to proteins, lipids and DNA in the remodeled heart. The primary finding of our study is that lipid damage is a good marker of cardiac oxidative damage in this model of tobacco smoke exposure. At least, after 2 months of tobacco smoke exposure, there were no differences in markers of protein or DNA damage.

Tobacco smoke can lead to cardiovascular diseases due to its effects on the vascular system and direct effects on cardiac remodeling and function [2–7]. Several studies have shown that ETS induces myocardial hypertrophy and ventricular dysfunction [2–9]. In our study, as expected, ETS induced myocyte hypertrophy, a hallmark of the cardiac remodeling process. Isolated hearts from animals exposed to tobacco smoke did not differ from hearts isolated from unexposed controls. In contrast, diastolic and systolic dysfunction were observed when cardiac function was assessed by echocardiogram [2–6]. It is important to note that preload, afterload and heart rate were controlled in our isolated heart model. Therefore, isolated heart preparation permits the analysis of cardiac function without interference of confounding variables, such as neurohormonal and hemodynamic factors. In this model, ETS induced hemodynamic alterations [35, 36]. Additionally, the cardiac remodeling and increased blood pressure induced by ETS were attenuated by lisinopril or propranolol [35, 36]. Therefore, the contradictory results of the in vivo and in vitro functional analyses reinforce the importance of neurohormonal/hemodynamic changes in the remodeling process induced by ETS.

In addition to neurohormonal changes, tobacco smoke also induces cardiac and systemic oxidative stress. In fact, previous studies have suggested that ETS increases NADPH oxidase activity and affects mitochondrial respiration, increasing ROS formation [5, 6]. Rafacho et al. also showed that after two months of ETS there was a decrease in the cardiac activity of antioxidant enzymes and an increase in LH [5]. Notably, supplementation with pentoxifylline, retinoic acid and beta-carotene, substances with antioxidant properties, have been shown to attenuate the ventricular remodeling process induced by smoking [37–40]. However, despite all the evidence showing the major role of ETS in cardiac oxidative damage, no study, until now, has analyzed the participation of lipid peroxidation, protein oxidation and DNA damage as targets of oxidative stress induced by exposure to cigarette smoke in this model.

ROS causes oxidative damage to DNA, proteins and lipids, and some studies have shown different behaviors of these oxidative macromolecular products. The time of their measurement, tissue specificity and the nature of ROS could explain these differences. It is well-known that protein carbonyl groups are formed early and are more stable than lipid peroxidation products, which are detoxified within minutes [24, 25]. Carbonyl groups have been analyzed as a biomarker of oxidative damage of proteins. Reznick et al. showed that in vitro exposure of plasma to cigarette smoke leads to rapid accumulation of protein carbonyl groups [41]. However, Yeh et al. showed that in 542 participants of the Early Lung Cancer Action Project carbonyl levels were not significantly different between current and former smokers and were not correlated with urine cotinine levels, pack-years or pack/day [19]. In our study, LV protein carbonyl concentrations did not differ between the control and ETS groups, assessed after two months of follow-up. This result suggests that at least at this time point, protein damage does not play a role in the cardiac remodeling induced by smoking.

Regarding DNA damage, several studies reported increased levels of 8-hydroxyguanine in smokers compared to nonsmokers [17–19]. However, conflicting results have been reported with comet assays to measure DNA damage related to ETS [20–22]. Hoffmann and Speit showed no significant difference in genotoxic effects in peripheral bloods cells between heavy smokers and nonsmokers [21]. In addition, an experimental study compared DNA damage in peripheral blood leukocytes from diabetic and non-diabetic female Wistar rats exposed to air or to cigarette smoke [20]. In this study, non-diabetic and diabetic rats exposed to cigarette smoke presented non-significant increases in DNA damage levels compared to the control group [20]. In our study, we evaluated peripheral blood leukocytes and cardiac tissue and we found no differences in comet tail intensities between the groups, even when the LV samples were exposed to FPG and endo III, which recognize oxidized purines and pyrimidines, respectively. Therefore, protein damage does not appear to participate in the chronic remodeling process in this model.

We should consider some limitations of our study. Firstly, we did not measure blood pressure and inflammatory mediators of the animals. In addition, we did not evaluate the lung tissue in this study.

Considering all of our results, we infer that LH was the only biomarker of oxidative stress that was increased in rats ETS. Therefore, at least following 2 months of ETS, we showed, by the first time, that lipid peroxidation seems more important than DNA or protein damage on cardiac remodeling induced by smoking.

## Conclusions

In conclusion, our results suggest that, in this model, lipid damage is a good marker of oxidative damage during the cardiac remodeling process induced by 2 months of tobacco smoke exposure.

## Abbreviations

+dP/dt: Maximum left ventricular pressure development rate
AP: Apurinic/apyrimidinic
BW: Body weigth
C: Control
CSA: Myocyte cross-sectional area
-dP/dt: The maximum left ventricular pressure decrease rate
ETS: Exposed to tobacco smoke
FPG: Formamidopyrimidine glycosylase
HE: Hematoxylin and eosin
LH: Lipid hydroperoxide
LV: Left ventricle
NADPH: Nicotinamide adenine dinucleotide phosphate
ROS: Reactive oxygen species
SBs: Labile sites
